# Supplementary material for: In silico prediction of candidate gene targets for the management of African cassava whitefly (Bemisia tabaci, SSA1-SG1), a key vector of viruses causing cassava brown streak disease
Source: PeerJ. 2024 Feb 23;12:e16949. doi: 10.7717/peerj.16949 (PMC10896082; doi:10.7717/peerj.16949)
Supplement: Supplemental Information 1 [file peerj-12-16949-s001.docx]

**SiRNA-Finder offtarget predictor results for the dsRNA sequences against *AQP1* and *SUC1* targets reported by Vyas *et al.,* 2017**

[**https://doi.org/10.1371/journal.pone.0168921**](https://doi.org/10.1371/journal.pone.0168921)

**AQP1 sequence used in this study to synthesize dsRNA (from accession # KF377800.1)**

tcgcacaatgccttggagccatctgtggagcaatcattctgaatgaaatcacgccaaaaacaggttacacggctgctggtaatctgggagtaacgacactgtctacaggagtttccgacctgcagggtgtggcgatagaagcactaatcacatttgtgctgcttttagttgtccagtccgtctgcgatgggaagcggaccgacatcaaaggatctatcggcgttgcgataggattcgcaattgct

**AGLU1 sequence used in this study to synthesize dsRNA (from accession # KF377803.1)**

ctgtccatccaaccctggattgccttttggtaatctttggcgggagagcgaccgctcacgtgcgtaataaagaagaaattgaatggcatatgggctcctggtttcccctcaaactgataatagtccattgttctatcgagagttgtatatgcttctgtcattagtacttttgttttcccctctttctttgtgtagaaatcgaagacctccctgaaccttgttatcaatctatatgtattcggttggtccatcgtccgcgaccggttgtaattccagtagttcgtcgggtcgagatcgggcgagagcaattcttggtctcgccatt

**Using MEAM1 genome database (containing all coding sequences of MEAM1)**

siRNA-Finder result for KF377800.1, a sequence for dsRNA against *AQP1* yielded one target Bta01973, an aquaporin 1 gene in MEAM1


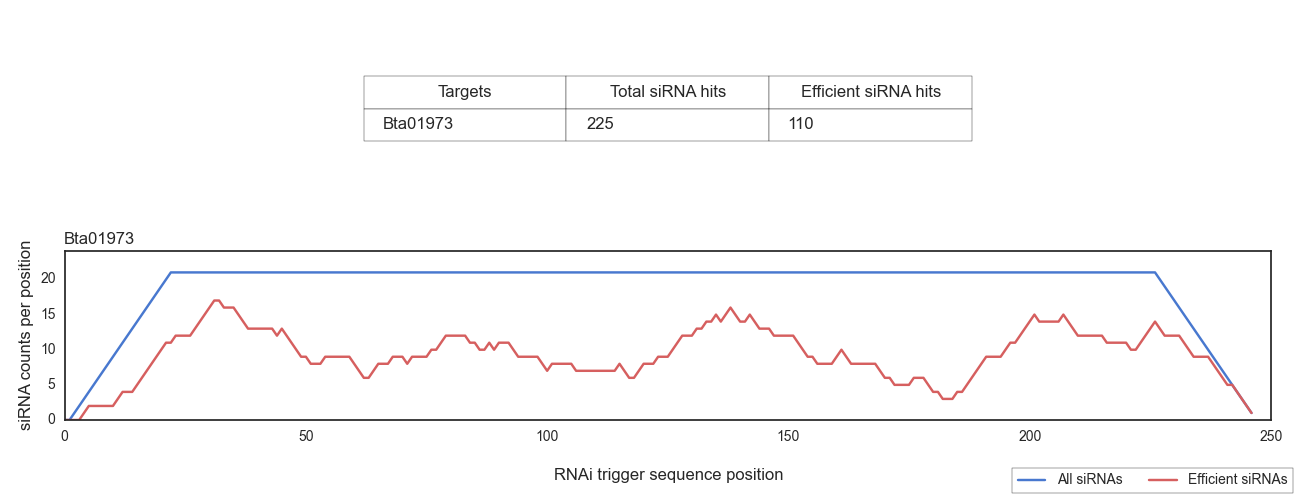


**Figure 1: siRNA-Finder result for KF377800.1, a sequence for dsRNA against *AQP1***

The AGLU1 sequence from accession KF377803.1 that was used to synthesize dsRNA against *SUC1* yielded no target when scanned against the MEAM1 genome database created in siRNA-Finder offtarget predictor software, containing all coding sequences for MEAM1 whitefly species.

However, the full sequence of KF377803.1 yielded two hits Bta10022 and Bta07452, which according to SignalP V4 had no signal peptide with D-Score of 0.369 and 0.416 respectively. However, this sequence had only 141 efficient siRNA hits onto target Bta07452


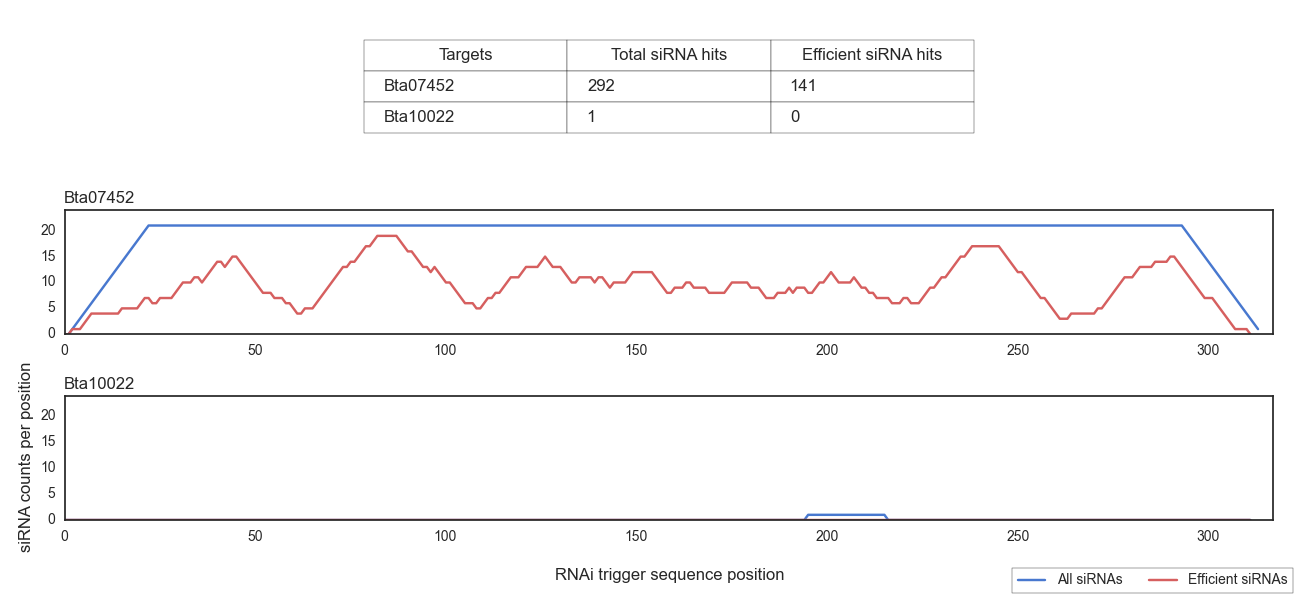


**Figure 2: Full sequence of KF377803.1 yielded two hits Bta10022 and Bta07452, which according to SignalP V4 had no signal peptide with D-Score of 0.369 and 0.416 respectively**

**Using SSA1-SG1 genome database**


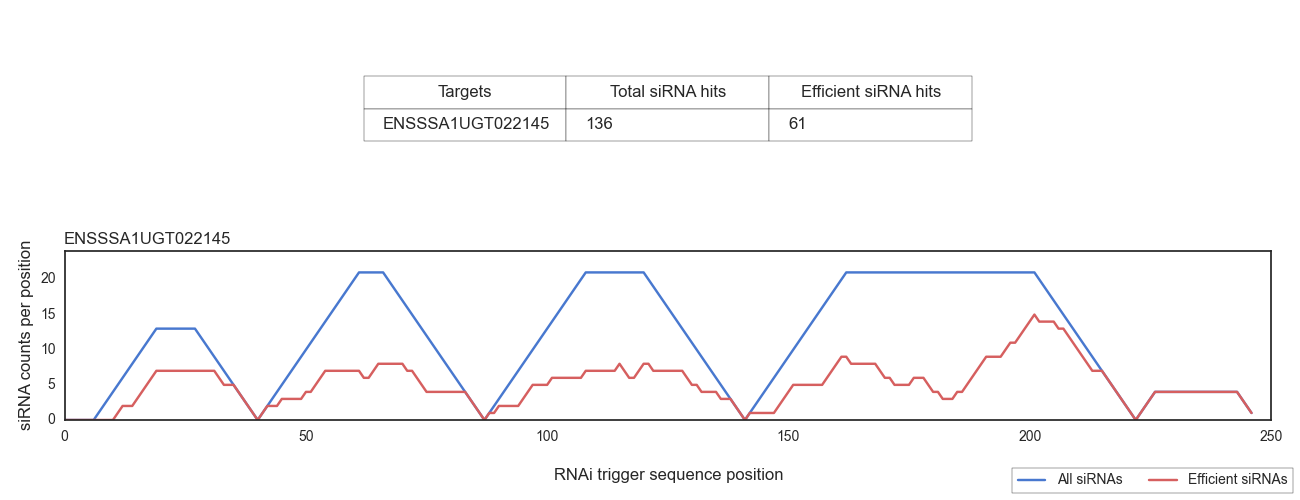


**Figure 3: siRNA-Finder result for KF377800.1, a sequence for dsRNA against *AQP1* yielded one target ENSSSA1UGT022145, an aquaporin 1 gene in SSA1-SG1**

The AGLU1 sequence from accession KF377803.1 that was used to synthesize dsRNA against *SUC1* yielded no target when scanned against the SSA1-SG1 genome database created in siRNA-Finder offtarget predictor software, containing all coding sequences for SSA1-SG1 whitefly species.

**siRNA-Finder offtarget predictor results for the dsRNA sequences against *AQP1* and *SUC1* targets for SSA1-SG1 whitefly species using SSA1-SG1 genome database (containing all the coding sequences of SSA1-SG1)**

***AQP1* sequence used in this study to synthesize dsRNA against AQP1_SSA1-SG1**

CCGATATTCGAGATGGGCCCACTCTCACAAAATGCATAGTTGCTGAGTTCGTTGGGACTTTGCTGTTAGTGCTCATAGGATGCATGTCGGTAGCATTTGTCCATCAGGACAACTTCGTTGACGTTGTGAAAATTGCCATGGCTTTCGGGCTCATCATCGCCTCTATGGTCCAGGCAATAGGTCACGTTAGTGGTTGTCACATCAATCCGGCTGTAACTTGTGGACTAGCTGTGTCGGGACATGTTAGCATAATAAAAGGTATGCTGTACATTGTCGCGCAATGCCTTGGAGCCATCTGTGGAGCAATCATTTTGAATGAAATCACGCCAAAAACAGGTTACACGGCTGCTGGTAAT


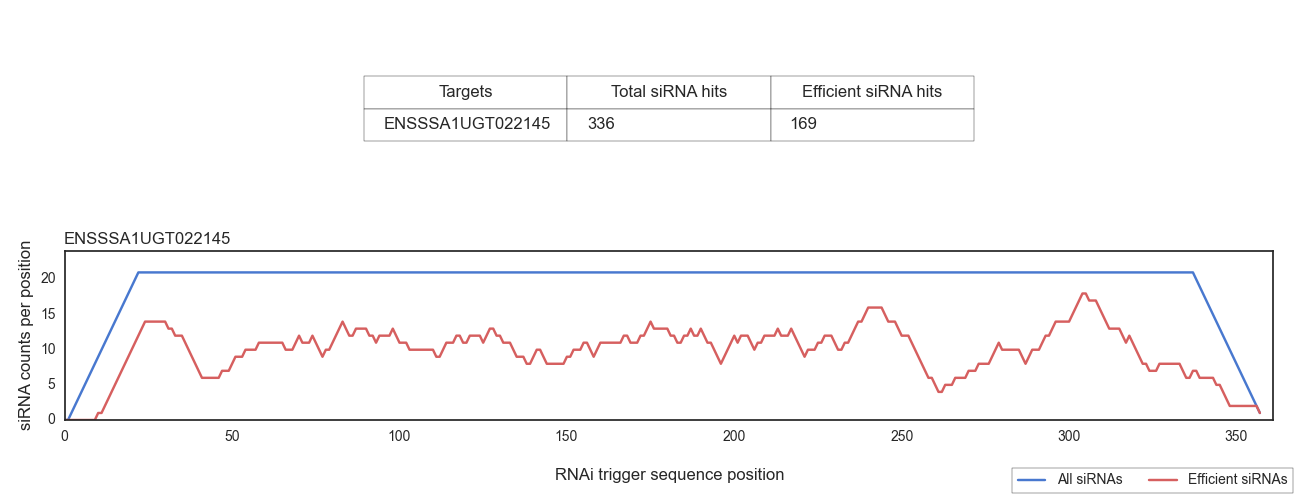


**Figure 4: The sequence used to synthesize dsRNA against *AQP1* yielded only one target, ENSSSA1UGTO22145, an aquaporin 1 in SSA1-SG1.**

***SUC1* sequence used in this study to synthesize dsRNA against SUC1_SSA1-SG1**

TACCCGCGATCATTCAAAGACTCGGATGGCGACGGAGTCGGCGACTTGAAAGGAATTGCAGAGAAAATTGATTACCTATCAAAACTAGGCGTCGAAGCAGTTTGGATTTCTCCGATTTTTCGCTCCCCGATGGCAGATTTTGGTTACGATATATCGGATTTCAGAGCAATCGAGCCAATGTTTGGCACTATGGAAGACTTTGAAAGGCTGAAAAGATTATTCCATAAAAATGGATTAAAAATGATTCTTGACTTCGTGCCAAACCATACCAGTGACGAACATGATTGGTTTAAGAAATCAGTCGCAAAAGTTGATCCGTACACAGATTATTACAACTGGGTCAACGGGAAGATGAG


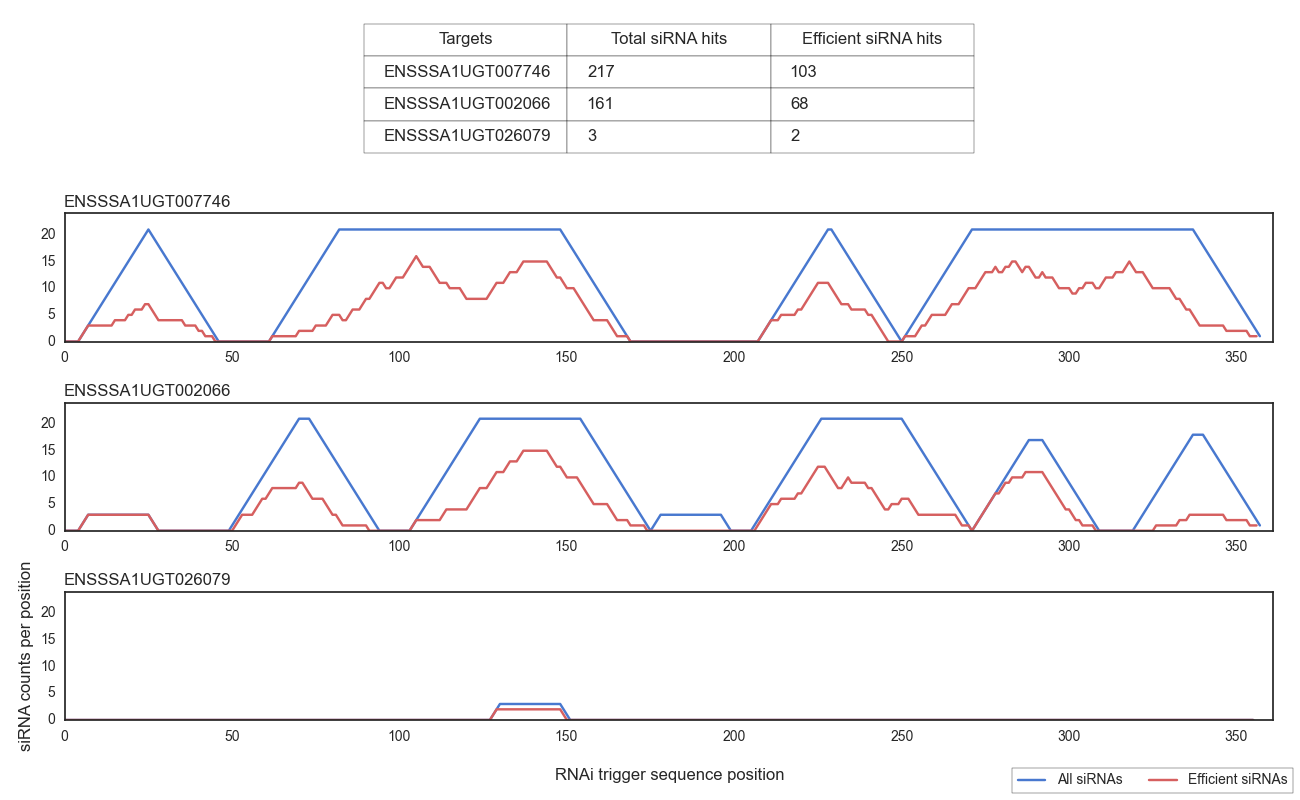


**Figure 5: The sequence used to synthesize dsRNA against SUC1 yielded only three target, ENSSSA1UGT007746, ENSSSA1UGT026079 and ENSSSA1UGT02066. The latter has been reported in this study as a candidate gene target for downregulating SUC1 in SSA1-SG1. Both ENSSSA1UGT007746 and ENSSSA1UGT02066 are highly homologous to Bta03818, an alpha-glucosidase in MEAM1.**
