# Supplementary material for: In silico prediction of candidate gene targets for the management of African cassava whitefly (Bemisia tabaci, SSA1-SG1), a key vector of viruses causing cassava brown streak disease
Source: PeerJ. 2024 Feb 23;12:e16949. doi: 10.7717/peerj.16949 (PMC10896082; doi:10.7717/peerj.16949)
Supplement: Supplemental Information 2 [file peerj-12-16949-s002.docx]

***In silico* prediction of candidate gene targets for the management of African cassava whitefly (*Bemisia tabaci,* SSA1-SG1-UG), the key vector of Cassava brown streak disease causative viruses**

Tadeo Kaweesi^1,2, 5*^, John Colvin^1^, Lahcen Campbell^3^, Paul Visendi^4^, Gareth Maslen^3^, Titus Alicai^5^ and Susan E Seal^1^


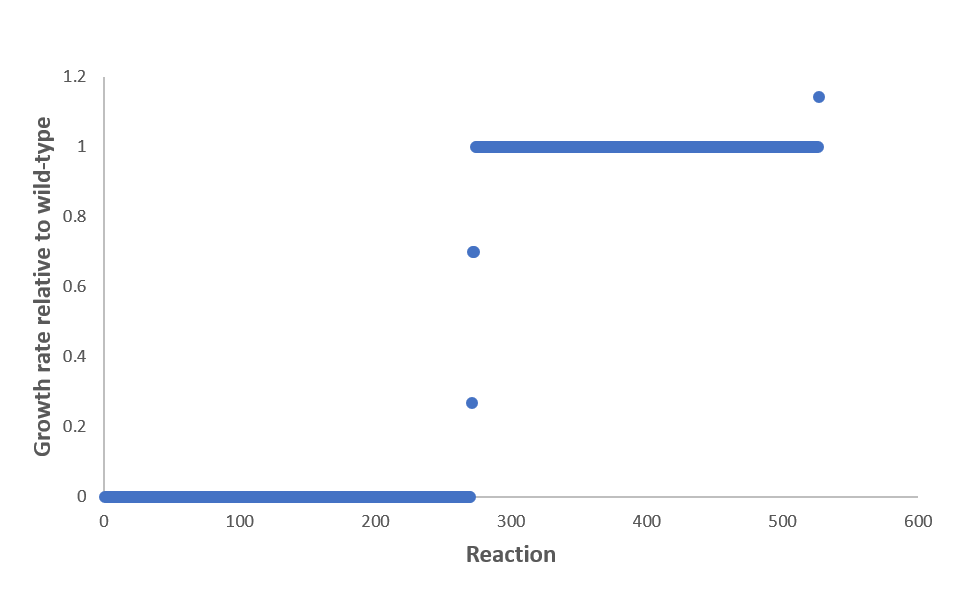


**Figure S1: Robustness of the metabolic network “SSA1-SG1_*Portiera*” iKT420 showing the effect of single reaction deletion on the growth rate of *B. tabaci* SSA1-SG1 and *Portiera* mutant relative to the wild-type, simulated using flux balance analysis. A total of 270 reaction deletions affected growth, therefore, were deemed indispensable/essential for the growth and survival of cassava *B. tabaci* SSA1-SG1**

**Table S1: Comparison of genomic features of *Portiera* genome from MEAM1, MED and SSA1-SG1**

| **Description** | **MEAM1** | **MED** | **SSA1-SG1** |
| --- | --- | --- | --- |
| Size (bp) | 352068 | 357461 | 347165 |
| GC content (%) | 26.2 | 26.1 | 26.2 |
| Number of coding sequences | 282 | 290 | 291 |
| Number of subsystems | 66 | 71 | 65 |
| Number of RNAs | 36 | 36 | 36 |
| Number of unique hypothetical proteins | 23* | 27** | 31*** |

*present in MEAM1 but missing in SSA1-SG1 **present in MED but missing in SSA1-SG1 ***present in SSA1-SG1 but missing in MEAM1

**Table S2: Missing protein/function in *Portiera* genome from MEAM1, MED and SSA1-SG1**

| **Unique protein/function** | **MEAM1** | **MED** | **SSA1-SG1** |
| --- | --- | --- | --- |
| 4-hydroxy-tetrahydrodipicolinate reductase (EC 1.17.1.8) | + | + | - |
| Inner membrane protein translocase component (YidC) | - | + | - |
| tRNA uridine 5-carboxymethylaminomethyl enzyme (GidA) | - | + | - |
| Cytochrome O ubiquinol oxidase subunit 1 | + | + | - |
| GTPase and tRNA-U34 5-formylation enzyme (TrmE) | - | + | - |
| SSU ribosomal protein S6p | - | + | - |


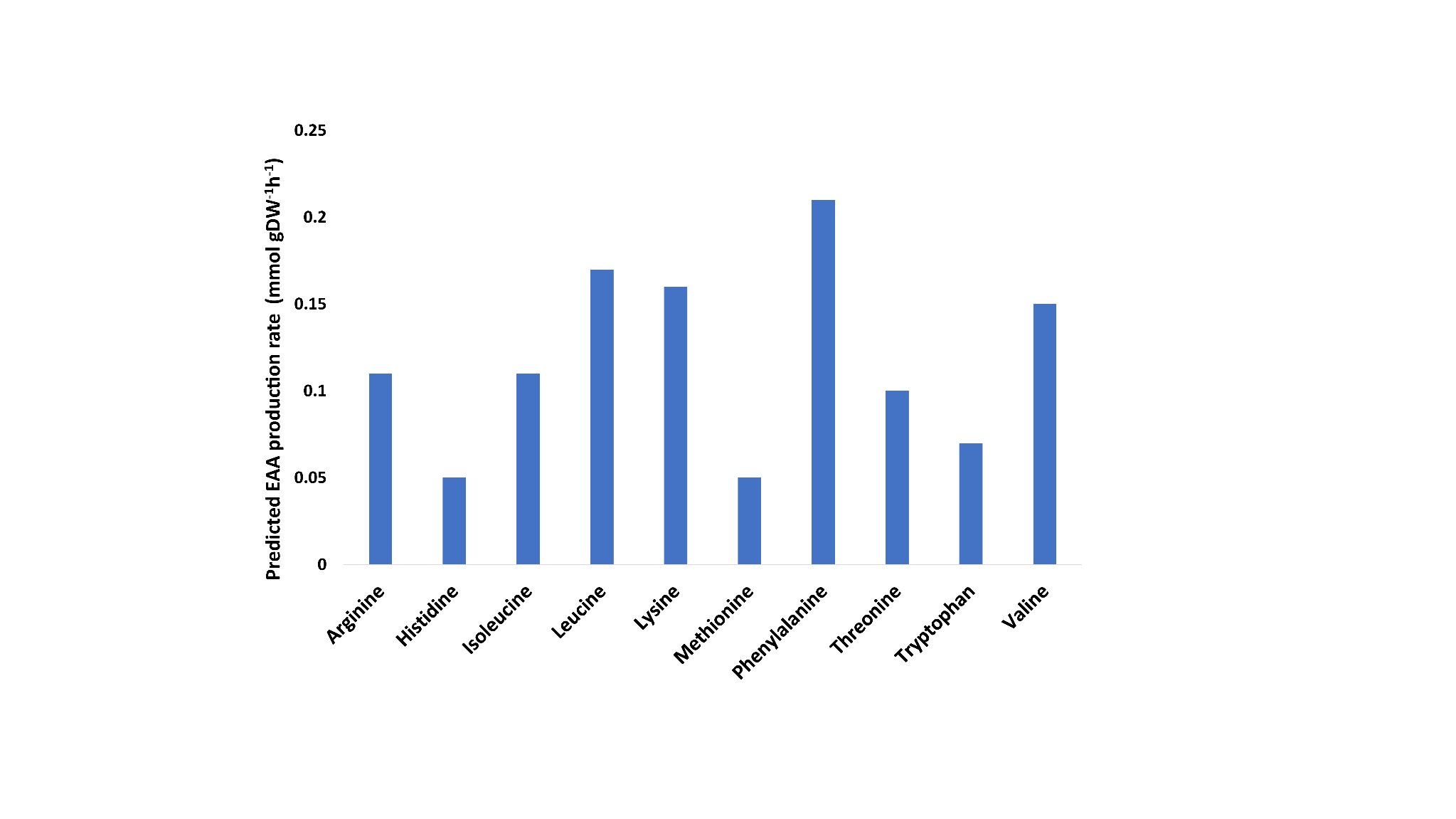


**Figure S2:** *In* *silico* prediction of production rates of essential amino acid of the two-compartment metabolic model of *B. tabaci* SSA1-SG1 and *Portiera.*

**Table S4: Comparison of genome-scale metabolic models of *B. tabaci* SSA1-SG1 and MEAM1**

| **Description** | ***B. tabaci* SSA1-SG1** | | |  | | ***B. tabaci* MEAM1** | | |
| --- | --- | --- | --- | --- | --- | --- | --- | --- |
|  | ***i*KT90** | ***i*KT330** | ***i*KT420**** | |  | ***i*NA94** | ***i*NA332** | ***i*NA774***** |
| Number of metabolic genes | 90 | 330 | 420 | |  | 94 | 332 | 774 |
| Number of intracellular reactions | 76 | 233 | 310 | |  | 76 | 236 | 774 |
| Number of metabolites | 150 | 251 | 402 | |  | 148 | 253 | 550 |
| Flux balance optimal solution (h^-1^) | 6.70 | 25.38 | 0.39 | |  | 12.02 | 26.55 | 0.20 |
| Total protein (mmol/gDw) |  |  | 6.20 | |  |  |  | 6.20 |
| Total cost for protein synthesis |  |  | 26.71 | |  |  |  | 26.71 |

** Two compartment model for *B. tabaci* SSA1-SG1 & Portiera *** Three-compartment (Ankrah *et al*., 2017)

**Table S5: List of essential reactions in a two-compartment metabolic model of SSA1-SG1**

| **Reaction Flux (mmol gDW^-1^ h^-1^)** | **Reaction Flux (mmol gDW^-1^ h^-1^)** |
| --- | --- |
| \| SSA1_Por_OCBT \| 0.111152 \| \| --- \| --- \| \| SSA1_Por_METS \| 0.046402 \| \| SSA1_Por_PRPPS \| 0.116569 \| \| SSA1_Por_ATPPRT \| 0.049133 \| \| SSA1_Por_PRATPP \| 0.049133 \| \| SSA1_Por_PRAMPC \| 0.049133 \| \| SSA1_Por_PRMICI \| 0.049133 \| \| SSA1_Por_IG3PS \| 0.049133 \| \| SSA1_Por_IGPDH \| 0.049133 \| \| SSA1_Por_HSTPT \| 0.049133 \| \| SSA1_Por_ASPK \| 0.260487 \| \| SSA1_Por_ASAD \| 0.260487 \| \| SSA1_Por_DHDPS \| 0.159891 \| \| SSA1_Por_THDPS \| 0.159891 \| \| SSA1_Por_SDPTA \| 0.159891 \| \| SSA1_Por_SDPDS \| 0.159891 \| \| SSA1_Por_HSD \| 0.100596 \| \| SSA1_Por_HSK \| 0.100596 \| \| SSA1_Por_THRS \| 0.100596 \| \| SSA1_Por_DDPA \| 0.277102 \| \| SSA1_Por_DHQS \| 0.277102 \| \| SSA1_Por_DHQTi \| 0.277102 \| \| SSA1_Por_SHK3Dr \| 0.277102 \| \| SSA1_Por_SHKK \| 0.277102 \| \| SSA1_Por_PSCVT \| 0.277102 \| \| SSA1_Por_CHORS \| 0.277102 \| \| SSA1_Por_ANS \| 0.067436 \| \| SSA1_Por_ANPRT \| 0.067436 \| \| SSA1_Por_PRAIi \| 0.067436 \| \| SSA1_Por_IGPS \| 0.067436 \| \| SSA1_Por_PPNDH \| 0.209666 \| \| SSA1_Por_ACHBS \| 0.113294 \| \| SSA1_Por_KARA2 \| 0.113294 \| \| SSA1_Por_DHAD2 \| 0.113294 \| \| SSA1_Por_ACLS \| 0.310706 \| \| SSA1_Por_KARA1 \| 0.310706 \| \| SSA1_Por_DHAD1 \| 0.310706 \| \| SSA1_Por_IPPS \| 0.16531 \| \| SSA1_Por_IPPMIb \| 0.16531 \| \| SSA1_Por_IPPMIa \| 0.16531 \| \| SSA1_Por_IPMD \| 0.16531 \| | \| SSA1_Por_OMCDC \| 0.16531 \| \| --- \| --- \| \| SSA1_Por_PDH \| 0.165312 \| \| SSA1_Por_AKGDH \| 0.209024 \| \| SSA1_Por_ATPS4rpp \| 3.89506 \| \| SSA1_Por_CYTBO3_4pp \| 0.493353 \| \| SSA1_Por_NADH16pp \| 0.493353 \| \| SSA1_Por_PSY1 \| 4.93E-06 \| \| SSA1_Por_PSY2 \| 4.93E-06 \| \| SSA1_Por_PDS1 \| 4.93E-06 \| \| SSA1_Por_PDS2 \| 4.93E-06 \| \| SSA1_Por_ZCAROTDH1 \| 4.93E-06 \| \| SSA1_Por_ZCAROTDH2 \| 2.47E-06 \| \| SSA1_Por_LCYBZC \| 2.47E-06 \| \| SSA1_Por_HEMEOS \| 2.47E-06 \| \| SSA1_Por_LIPOS \| 2.47E-06 \| \| SSA1_Por_MTHFR2 \| 0.046402 \| \| SSA1_Por_DPR \| 2.91E-06 \| \| SSA1_Por_CLPNS160pp \| 0.000318 \| \| SSA1_Por_DM_LIPOPB \| 2.47E-06 \| \| SSA1_Por_DM_GLYC \| 0.000318 \| \| SSA1_Por_O2tex \| 0.246684 \| \| SSA1_Por_H2Otex \| -2.83424 \| \| SSA1_Por_CO2tcy \| 1.77185 \| \| SSA1_Por_UP_2DHP \| 2.91E-06 \| \| SSA1_Por_UP_PYR \| 0.992473 \| \| SSA1_Por_UP_2OBUT \| 0.113294 \| \| SSA1_Por_UP_E4P \| 0.277102 \| \| SSA1_Por_UP_PEP \| 0.554204 \| \| SSA1_Por_UP_R5P \| 0.116569 \| \| SSA1_Por_UP_PG160 \| 0.000636 \| \| SSA1_Por_UP_GGDP \| 9.87E-06 \| \| SSA1_Por_UP_FRDP \| 2.47E-06 \| \| SSA1_Por_UP_OCTAPB \| 2.47E-06 \| \| SSA1_Por_UP_PYDX5P \| 2.47E-06 \| \| SSA1_Por_UP_THMPP \| 2.47E-06 \| \| SSA1_Por_UP_FMNH2 \| 2.47E-06 \| \| SSA1_Por_UP_THDP \| 0.159891 \| \| SSA1_Por_UP_MLTHF \| 0.046402 \| \| SSA1_Por_UP_COA \| 0.049135 \| \| SSA1_Por_UP_PHEME \| 2.47E-06 \| \| SSA1_Por_UP_NADP \| 2.61E-05 \| |

**Table S2: Continued**

| **Reaction Flux (mmol gDW^-1^ h^-1^)** | **Reaction Flux (mmol gDW^-1^ h^-1^)** |
| --- | --- |
| \| SSA1_Por_UP_NADH \| 0.000109 \| \| --- \| --- \| \| SSA1_Por_UP_DATP \| 0.001809 \| \| SSA1_Por_UP_DCTP \| 0.000643 \| \| SSA1_Por_UP_DGTP \| 0.000643 \| \| SSA1_Por_UP_DTTP \| 0.001809 \| \| SSA1_Por_UP_ADP \| 0.255983 \| \| SSA1_Por_UP_CTP \| 0.006216 \| \| SSA1_Por_UP_GTP \| 0.010014 \| \| SSA1_Por_UP_UTP \| 0.006709 \| \| SSA1_Por_UP_AMET \| 4.93E-06 \| \| SSA1_Por_UP_HCYS-L \| 0.046402 \| \| SSA1_Por_UP_ORN \| 0.111152 \| \| SSA1_Por_UP_ASN-L \| 0.013436 \| \| SSA1_Por_UP_ASP-L \| 0.356567 \| \| SSA1_Por_UP_CYS-L \| 0.017419 \| \| SSA1_Por_UP_GLN-L \| 0.235647 \| \| SSA1_Por_UP_GLY \| 0.015866 \| \| SSA1_Por_UP_PRO-L \| 0.007604 \| \| SSA1_Por_UP_SER-L \| 0.083706 \| \| SSA1_Por_UP_TYR-L \| 0.006368 \| \| SSA1_Por_UP_ARG-L \| 0.010652 \| \| SSA1_Por_UP_HIS-L \| 0.004045 \| \| SSA1_Por_UP_ILE-L \| 0.021221 \| \| SSA1_Por_UP_LEU-L \| 0.02112 \| \| SSA1_Por_UP_LYS-L \| 0.021646 \| \| SSA1_Por_UP_PHE-L \| 0.007694 \| \| SSA1_Por_UP_VAL-L \| 0.01781 \| \| SSA1_Por_EF_SUCC \| 0.159891 \| \| SSA1_Por_EF_G3P \| 0.067436 \| \| SSA1_Por_EF_PPI \| 0.283837 \| \| SSA1_Por_EF_PANT-R \| 2.91E-06 \| \| SSA1_Por_EF_PHPYR \| 0.209666 \| \| SSA1_Por_EF_3MOB \| 0.145396 \| \| SSA1_Por_EF_3MOP \| 0.113294 \| \| SSA1_Por_EF_4MOP \| 0.16531 \| \| SSA1_Por_EF_SUCCOA \| 0.049133 \| \| SSA1_Por_EF_DAD-5 \| 4.93E-06 \| \| SSA1_Por_EF_AMP \| 0.198622 \| \| SSA1_Por_EF_THF \| 0.046402 \| \| SSA1_Por_EF_AICAR \| 0.049133 \| \| SSA1_Por_EF_23DHDP \| 0.159891 \| | \| SSA1_Por_EF_26DAP-LL \| \| 0.159891 \| \| --- \| --- \| --- \| \| SSA1_Por_EF_ARGSUC \| \| 0.082103 \| \| SSA1_Por_EF_HISP \| \| 0.049133 \| \| SSA1_Por_EF_TRP-L \| \| 0.065903 \| \| SSA1_Por_EF_THR-L \| \| 0.088147 \| \| SSA1_Por_EF_MET-L \| \| 0.039207 \| \| SSA1_Bt_ASPTA \| \| 0.817173 \| \| SSA1_Bt_ASNS1 \| \| 0.135612 \| \| SSA1_Bt_DHDPRy \| \| 0.159891 \| \| SSA1_Bt_DAPE \| 0.159891 \| \| \| SSA1_Bt_DAPDC \| 0.159891 \| \| \| SSA1_Bt_GLNS \| 0.842491 \| \| \| SSA1_Bt_CBPS \| 0.015376 \| \| \| SSA1_Bt_P5CR \| 998.112 \| \| \| SSA1_Bt_P5CDr \| 0.217739 \| \| \| SSA1_Bt_GSC \| 0.111152 \| \| \| SSA1_Bt_ORNTAr \| 0.111152 \| \| \| SSA1_Bt_ARGSL \| 0.111152 \| \| \| SSA1_Bt_METAT \| 4.93E-06 \| \| \| SSA1_Bt_CYSTS \| 0.113294 \| \| \| SSA1_Bt_CYSTGL \| 0.113294 \| \| \| SSA1_Bt_HISTP \| 0.049133 \| \| \| SSA1_Bt_HISTD \| 0.049133 \| \| \| SSA1_Bt_ILETA \| 0.113294 \| \| \| SSA1_Bt_LEUTAi \| 0.16531 \| \| \| SSA1_Bt_PHETA1 \| 0.209666 \| \| \| SSA1_Bt_PHETHPTOX \| 0.104398 \| \| \| SSA1_Bt_VALTA \| 0.145393 \| \| \| SSA1_Bt_GTPCI \| 0.104398 \| \| \| SSA1_Bt_PTHPS \| 0.104398 \| \| \| SSA1_Bt_SPR \| 0.104398 \| \| \| SSA1_Bt_GHMT2r \| 0.238214 \| \| \| SSA1_Bt_MTHFD \| 0.195203 \| \| \| SSA1_Bt_MTHFC \| 0.195203 \| \| \| SSA1_Bt_RBFK \| 2.47E-06 \| \| \| SSA1_Bt_ANNA \| 0.000135 \| \| \| SSA1_Bt_DNGAL \| 0.000135 \| \| \| SSA1_Bt_NADK \| 2.61E-05 \| \| \| SSA1_Bt_MOHMT \| 2.91E-06 \| \| \| SSA1_Bt_PANTS \| 2.91E-06 \| \| \| SSA1_Bt_PNTK \| 2.91E-06 \| \| |

**Table S2: Continued**

| **Reaction Flux (mmol gDW^-1^ h^-1^)** | **Reaction Flux (mmol gDW^-1^ h^-1^)** |
| --- | --- |
| \| SSA1_Bt_PPNCL2 \| 2.91E-06 \| \| --- \| --- \| \| SSA1_Bt_PPCDC \| 2.91E-06 \| \| SSA1_Bt_PTPATi \| 2.91E-06 \| \| SSA1_Bt_DPCOAK \| 2.91E-06 \| \| SSA1_Bt_ALASm \| 1.97E-05 \| \| SSA1_Bt_PPBNGS \| 9.87E-06 \| \| SSA1_Bt_HMBS \| 2.47E-06 \| \| SSA1_Bt_UPP3S \| 2.47E-06 \| \| SSA1_Bt_UPPDC1 \| 2.47E-06 \| \| SSA1_Bt_CPPPGO \| 2.47E-06 \| \| SSA1_Bt_PPPGO \| 2.47E-06 \| \| SSA1_Bt_FCLT \| 2.47E-06 \| \| SSA1_Bt_GLUK \| 1.88671 \| \| SSA1_Bt_GAPD \| 3.92792 \| \| SSA1_Bt_PGK \| 3.92792 \| \| SSA1_Bt_PGM \| 3.92792 \| \| SSA1_Bt_ENO \| 3.92792 \| \| SSA1_Bt_PYK \| 3.37372 \| \| SSA1_Bt_PDH \| 0.666063 \| \| SSA1_Bt_PCr \| 0.978394 \| \| SSA1_Bt_CS \| 0.665922 \| \| SSA1_Bt_ACONTa \| 0.665922 \| \| SSA1_Bt_ACONTb \| 0.665922 \| \| SSA1_Bt_SUCOAS \| 0.049113 \| \| SSA1_Bt_SUCDi \| 0.209004 \| \| SSA1_Bt_FUM \| 0.504702 \| \| SSA1_Bt_MDH \| 0.504702 \| \| SSA1_Bt_PGL \| 0.531102 \| \| SSA1_Bt_RPI \| 0.440854 \| \| SSA1_Bt_CYOO6m \| 1.66454 \| \| SSA1_Bt_CYOR(q9)m \| 3.32908 \| \| SSA1_Bt_TRDR \| 0.004903 \| \| SSA1_Bt_PPA \| 0.565679 \| \| SSA1_Bt_ACACT1r \| 4.69E-05 \| \| SSA1_Bt_HMGCOAS \| 4.69E-05 \| \| SSA1_Bt_HMGCOARx \| 4.69E-05 \| \| SSA1_Bt_MEVK1x \| 4.69E-05 \| \| SSA1_Bt_PMEVKx \| 4.69E-05 \| \| SSA1_Bt_DPMVDx \| 4.69E-05 \| \| SSA1_Bt_IPDDI \| 1.23E-05 \| \| SSA1_Bt_DMATT \| 1.23E-05 \| | \| SSA1_Bt_GRTT \| 1.23E-05 \| \| --- \| --- \| \| SSA1_Bt_FRTT \| 9.87E-06 \| \| SSA1_Bt_HCO3E \| 1.10492 \| \| SSA1_Bt_CAT \| 1.23E-06 \| \| SSA1_Bt_ASPCT \| 0.015376 \| \| SSA1_Bt_DHORTS \| 0.015376 \| \| SSA1_Bt_DHORD2 \| 0.015376 \| \| SSA1_Bt_ORPRT \| 0.015376 \| \| SSA1_Bt_ORPDC \| 0.015376 \| \| SSA1_Bt_UMPK \| 0.015376 \| \| SSA1_Bt_ATUD \| 0.013567 \| \| SSA1_Bt_ATCM \| 2.91E-06 \| \| SSA1_Bt_ATCD \| -0.00064 \| \| SSA1_Bt_ATDCD \| 0.000643 \| \| SSA1_Bt_ATDGD \| 0.000643 \| \| SSA1_Bt_ATGD \| 0.173724 \| \| SSA1_Bt_NDPK \| 0.001809 \| \| SSA1_Bt_ATDTD \| 0.001809 \| \| SSA1_Bt_PRDPAR \| 0.125234 \| \| SSA1_Bt_PPRGL \| 0.125234 \| \| SSA1_Bt_FPGFT \| 0.125234 \| \| SSA1_Bt_PRFGS \| 0.125234 \| \| SSA1_Bt_PRFGCL \| 0.125234 \| \| SSA1_Bt_PRAIC \| 0.125234 \| \| SSA1_Bt_PRAIS \| 0.125234 \| \| SSA1_Bt_AIAL \| 0.125234 \| \| SSA1_Bt_FPAIF \| 0.174367 \| \| SSA1_Bt_ICH \| 0.174367 \| \| SSA1_Bt_IMPD \| 0.115055 \| \| SSA1_Bt_GMPS(glu) \| 0.115055 \| \| SSA1_Bt_AGPT \| 0.115055 \| \| SSA1_Bt_DGOTO \| 0.000643 \| \| SSA1_Bt_ADSS \| 0.059312 \| \| SSA1_Bt_AAL(fum) \| 0.059312 \| \| SSA1_Bt_ATAM \| 0.537788 \| \| SSA1_Bt_DAOTO \| 0.001809 \| \| SSA1_Bt_DCDT \| 0.000643 \| \| SSA1_Bt_TMDS \| 0.001809 \| \| SSA1_Bt_DTMPK \| 0.001809 \| \| SSA1_Bt_DM_ALA-B \| 2.91E-06 \| \| SSA1_Bt_DM_PG160 \| 0.000636 \| |

**Table S2: Continued**

| **Reaction Flux (mmol gDW^-1^ h^-1^)** |  |
| --- | --- |
| \| SSA1_Bt_DM_OCTAPB \| 2.47E-06 \| \| --- \| --- \| \| SSA1_Bt_DM_DHBPT \| 0.104398 \| \| SSA1_Bt_O2tex \| 2.01563 \| \| SSA1_Bt_Pitex \| -0.34944 \| \| SSA1_Bt_NH4tex \| -3.69554 \| \| SSA1_Bt_UP_hcys-L \| 0.159695 \| \| SSA1_Bt_UP_thmpp \| 2.47E-06 \| \| SSA1_Bt_UP_ribflv \| 2.47E-06 \| \| SSA1_Bt_UP_pdx5p \| 2.47E-06 \| \| SSA1_Bt_UP_nicrnt \| 0.000135 \| \| SSA1_Bt_EF_dad-5 \| 4.93E-06 \| \| SSA1_Bt_EF_pppi \| 0.104398 \| \| Bt_GROW \| 0.312566 \| \| EX_pppi(e) \| 0.104398 \| \| EX_dad-5(e) \| 4.93E-06 \| \| EX_o2(e) \| -2.01563 \| \| EX_h(e) \| 3.43033 \| \| EX_pi(e) \| -0.34944 \| \| EX_nh4(e) \| -3.69554 \| \| EX_hcys-L(e) \| -0.1597 \| \| EX_thmpp(e) \| -2.47E-06 \| \| EX_ribflv(e) \| -2.47E-06 \| \| EX_pdx5p(e) \| -2.47E-06 \| \| EX_nicrnt(e) \| -0.00013 \| |  |

**Table S3:** **Primers used in the validation of gene expression of selected genes**

| **Target gene** | **Primer sequence** | **Amplicon length (bp)** | **Tm (^0^C)** |
| --- | --- | --- | --- |
| ENSSSA1UGT022145 (AQP1) | F - TTGTTTCGCAAGTTTGCCGT | 90 | 59.83 |
|  | R - GACTGATTGACGCCCTGGAT |  | 59.82 |
| ENSSSA1UGT002057 (SUC1) | F - AACACTGCGAATAGCGCATC | 86 | 59.35 |
|  | R - CGCCACTCTAGATGTTCGCA |  | 60.18 |
| ENSSSA1UGT002066 (SUC2) | F - CGGTAAGGTCTGAAACTGCGAT | 110 | 60.86 |
|  | R - GTTTTGCTAGATGTGCAAGGCA |  | 58.76 |
| Diaminopimelate decarboxylase (*LysA*) | F - ACTACAATTCTCGCCCTCGC | 86 | 60.18 |
|  | R - GTCATCAAAGGTCTCCCGCC |  | 60.74 |
| Arginosuccinate lyase (*ArgH*) | F - AAGCTCTGGTGTAAGGCACA | 98 | 59.24 |
|  | R - AGGATGTCTTGGGTCGCTTC |  | 59.75 |
| Branched-chain-amino-acid aminotransferase (*BCAT*) | F - CGTCCAGAGTCAGTGGCAA | 93 | 59.63 |
|  | R - GTTCATGGCTCCGGCTTCAG |  | 61.37 |
| Aspartate aminotransferase (*AAT*) | F - GGTCCTACCAGTTGTGCGAA | 95 | 59.97 |
|  | R - AGAATGGAGCCGAACCCAAG |  | 60.04 |
| 60S ribosomal protein L13a (*RPL13A*) | F - CATTCCACTACAGAGCTCCA | 101 | 60.00 |
|  | R - TTTCAGGTTTCGGATGGCTT |  | 60.00 |
| β-Tubulin (β-Tub) | F - TGTCAGGAGTAACGACGTGTTTG | 150 | 60.00 |
|  | R - TTCGGGAACGGTAAGTGCTC |  | 60.00 |
| 4-hydroxy-tetrahydrodipicolinate reductase (*dapB*) | F - TGGTAAAAGACTACCAGGCGAA | 118 | 59.37 |
|  | R - AGCTTGGTGTTTACAGCTGAGAG |  | 60.81 |


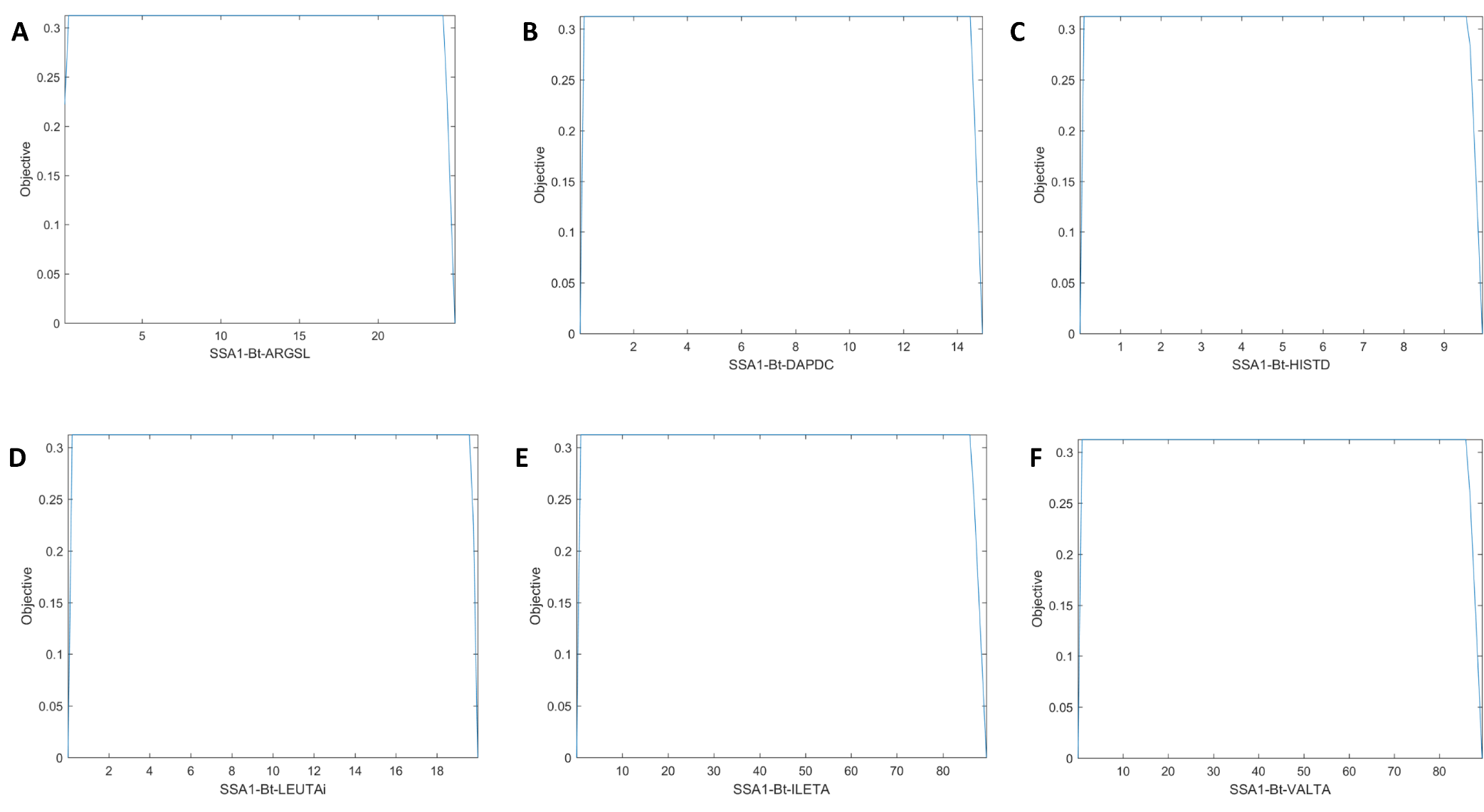


**Figure S2: Robustness analysis measuring sensitivity of the metabolic object function (growth rate of B. tabaci SSA1-SG1) to the quantitative flux levels through terminal reactions for (A) arginine biosynthesis, (B) lysine biosynthesis, (C) histidine biosynthesis, (D) leucine biosynthesis, (E) isoleucine synthesis and (F) valine biosynthesis.**


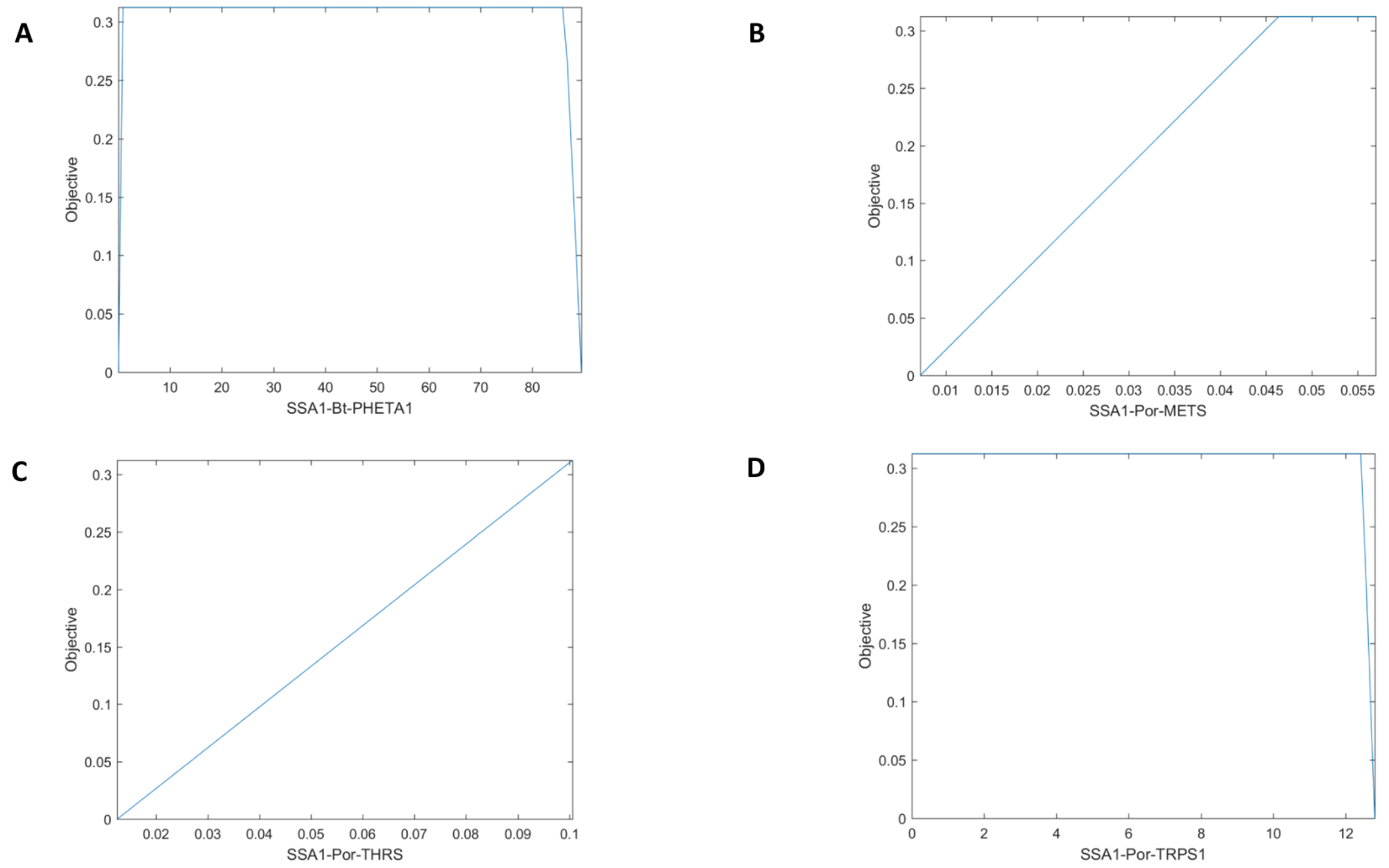


**Figure S3: Robustness analysis measuring sensitivity of the metabolic object function (growth rate of B. tabaci SSA1-SG1) to the quantitative flux levels through terminal reactions for (A) phenylalanine biosynthesis (B) methionine biosynthesis (C) threonine biosynthesis and (D) tryptophan biosynthesis**
